# Differences in regional distribution and inequality in health-resource allocation on institutions, beds, and workforce: a longitudinal study in China

**DOI:** 10.1186/s13690-021-00597-1

**Published:** 2021-05-17

**Authors:** Enhong Dong, Jie Xu, Xiaoting Sun, Ting Xu, Lufa Zhang, Tao Wang

**Affiliations:** 1 School of Nursing and Health Management, Shanghai University of Medicine & Health Science, 279 Zhouzhu Road, Pudong New District, Shanghai, 201318 China; 2 School of Media and Communication, Shanghai Jiao Tong University, Shanghai, 200240 China; 3 Emergency Department, Dezhou People’s Hospital, Dezhou, 253003 Shandong Province China; 4 Shanghai Tenth People’s Hospital Affiliated to Tongji University, Shanghai, 200072 China; 5 School of International and Public Affairs, Shanghai Jiao Tong University, Shanghai, 200030 China; 6 Department of Orthopaedics and Traumatology, Shanghai East Hospital Tongji University School of Medicine, Shanghai, 200127 China; 7 College of Arts and Media, Tongji University, Shanghai, 200092 China

**Keywords:** Health-care resources, Regional difference, Inequality, Central districts, Suburban districts

## Abstract

**Background:**

The distribution of health-care resources is foundational to achieving fairness and having access to health service. China and its local Shanghai’s government have implemented measures to allocate health-care resources with the equity as one of the major goals since 2009-health-care reform. The aim of this study was to analyze differences in regional distribution and inequality in health-resource allocation on institutions, beds, and workforce in Shanghai over 7 years.

**Methods:**

The study was conducted using 2010–2016 data to analyze health-resource allocation on institutions, beds, and workforce in Shanghai, China. The annual growth rate (AGR) was used to evaluate the time trends of health-care resource from 2010 to 2016, and Theil index was calculated to measure inequality of five indicators of health-care resource allocation during this study period.

**Results:**

All quantities of health-care resources per 1000 people increased across Shanghai districts from 2010 to 2016. Compared with suburban districts, the central districts had higher ratios on five health-care resource indicators, and faster average growth in the bed and nurse indicator. The Theil of the indicators, except for doctors in hospitals, all exhibited downward time trends.

**Conclusions:**

Regional difference between urban and rural areas and inequality between institution and workforce, especially for doctors, still existed. Some targeted measures including but not limited to income raising, facilitation of transportation conditions, investment of more fiscal funds, enhancement of health-care service provision for rural residents should be fully considered to narrow resource distribution gap between urban and rural districts and mitigate the inequality of health-care resource allocation.

**Supplementary Information:**

The online version contains supplementary material available at 10.1186/s13690-021-00597-1.

## Background

As a fundamental component of human right, equity is a basic need for health access and health care resource allocation fairness. Reasonable health-care resource allocation is in turn crucial to achieving health service equity, which contributes to public health outcomes and mitigates social conflict [1–3]. Since the distribution of health-care resources is a critical component of health-care access, in many countries, health-care reform was launched to improve the allocation of health-care resources to provide universal and equitable access to health care, which is recognized as a critical human right, for the equitable allocation of health-care resources helps deliver health-care resources to those most in need and ensures accessibility to basic health services as well as fairness for vulnerable populations [4]. Otherwise, inequality in health-care resources incurs adverse consequences, for example, the uneven distribution of health-care allocation could lead to growing inequalities between the rich and poor with respect to health and the economic burden of disease [5]. Many literatures have declared that highly accessible health care can play a crucial role in promoting regional health equity. Since 2009, aiming to provide households with secure, efficient, convenient, equitable, and affordable health-care services by reversing the market-oriented health system into one with universal benefits, China had launched a new round of health-care reform and strengthened the government’s role in health-care, its commitment to equity, and its willingness to experiment with regulated market approaches. In addition to genetic features, the Chinese health-care system also has some specific characteristics. Take the health financing system as a case, this system collects revenues from three main sources: government expenditure, social expenditure and out-of-pocket (OOP) payments according to the official classification. These revenues are distributed through the basic medical security system including Basic Medical Insurance (BMI) programs and Medical Financial Assistance (MFA) programs for the poor to cover urban and suburban residents in China. Under BMI, employees in urban areas are covered by Urban Employee Basic Medical Insurance (UEBMI), while unemployed residents in urban areas are covered by Urban Residents Basic Medical Insurance (URBMI) and residents in rural areas are covered by New Rural Cooperative Medical System (NRCMS). The MFA provides security for the poor in both urban and rural areas, helping them to enroll in basic medical insurance and to apply for extra reimbursement for medical expenses. The public health system, mainly financed by the government, provides basic public health services free of charge to all residents nationwide. Accordingly, since 2009, the Shanghai’s government has taken corresponding measures to re-allocate health-care resources between central districts and rural ones, conforming tightly to the national health reform strategies and guidelines and three tenets for Shanghai’s health-care reform (build a foundation, manage for the long term, and make reforms sustainable). For example, in 2009, shanghai government spent approximately $1 billion to carry out a policy named “5 + 3 + 1” to strengthen the role of public hospitals, and to deepen health-care reforms. Under the policy, nine tertiary hospitals in rural areas were constructed and a “1560” accessible radius to health care were formulated, so that patients in urban areas can walk to a nearest medical institution within 15 min, and patients in suburban districts can visit a tertiary hospital within 60 min by public transportation [6]. As a result of these measures, nine new tertiary hospitals were built and 6000 beds were provided in the outer rings of Shanghai. Rural patients no longer had to drive an hour or two to visit tertiary hospitals in the city center. With the increase number of institutions, beds and workforce transferring from “mother hospitals” to new hospitals, the distribution of health-care resources has become more balanced [7, 8]. However, many studies navigated variations of health-resource allocation quantity and inequality in China and have seen the widening urban–rural disparities in health-care resources across China [9–14], including the one conducted in Shanghai [15]. However, they have been unaware of the difference over time in health-resource allocation as well as its relation to China’s 2009 new health-care reform. Taking consideration of the overall goal of China’s new health-care guidelines and plans to promote more equitable and efficient health-care resource distribution, it is of importance to examine the differences in health-resource distribution and the inequity of allocation in Shanghai over time since the new reform.

Thereby, the object of this study was, firstly, to investigate regional difference in health-resource distribution and, secondly, to analyze the inequity of their allocation over 7 years (2010–2016) in Shanghai, in order to see whether both of them have experienced noticeable and sound changes after the new-round reform in China since 2009.

## Methods

### Data source

The data of the study was from the Shanghai Medical Statistical Yearbook (2010–2016) and the Shanghai Statistical Yearbook (2010–2016), published by the Shanghai Health Commission and Shanghai Statistics Bureau, respectively. The indicators used in the study included the number of health institutions, the number of beds, the number of technicians, the number of doctors, and the number of nurses. These data were selected from the Shanghai Medical Statistical Yearbook from 2010 to 2016. Table 1 provided all 5 indicators and interpreted their definitions along with how they were measured. Per capita measures of five indicators were calculated by dividing the annual population of the whole city and every administrative division into the absolute values of these indicators.

**Table 1:** Indicators of health-resource allocation, their definitions, and how they were measured

Indicator	Definition	How indicator was measured
Number of institutions	It refers to the institutions such as hospitals, primary health care institutions, professional public health agencies and other healthcare institutions which have obtained the legal registration certificates from the health administrative departments in China	Number of institutions divided by the population
Number of beds	It refers to the actual number of beds in medical institutions, including formal beds, simple beds, care beds, beds are being disinfected or repaired, not including neonatal beds, pre-delivery beds, observation beds, temporary beds and the accompany beds for patients’ family	Number of beds divided by the population
Number of technicians	It refers to the workforce who assist medical staff complete tasks around their assigned unit or clinic and accommodate patient needs, including pharmacists and radiologists; registered nurses were excluded	Number of technicians in hospitals divided by the population
Number of doctors	It refers to the physicians who hold a practicing physician certificate, including practicing physicians and assistants in China. Those who are engaged in the management of health workers as part of the health workforce, such as presidents, vice presidents, and party secretaries were excluded	Number of doctors divided by the population
Number of nurses	It refers to registered nurses who have obtained the legal practicing nurses’ certificates. Those who engaged in the management of health workers are not included as health workforce, such as president, vice president, party secretary	Number of nurses divided by the population

Shanghai is one of four municipalities under the direct control of the state council of China, and it is divided into 16 districts, including seven urban and nine suburban districts. Shanghai’s urban administrative districts are Huangpu, Xuhui, Changning, Jing’an, Putuo, Hongkou, and Yangpu as well as its rural administrative ones: Baoshan, Jiading, Jinshan, Minhang, Songjiang, Qingpu, Fengxian, Pudong New Area and Chongming. During 7 years (2010 to 2016), Shanghai had experienced three administration division mergers to facilitate sustainable development of all the districts involved, so as to enhance the administrative efficiency of urban function and resource distribution for the city, and reduce administrative costs. Particularly, Luwan District was merged into its neighbor district, forming the new Huangpu District in 2011. Zhabei District was merged with Jing’an District in 2015; and Chongming County was upgraded from county level to district level named Chongming District in 2016. In order to maintain data comparability, the new data of the 16 administration divisions were formatted by integrating the data of the two merged Luwan and Zhabei districts into those of Huangpu and Jing’an, respectively.

Since using secondary data, the study did not require patient or public involvement.

### Data analysis

The annual growth rates (AGRs) of the five types of health-care resource were also calculated from 2010 to 2016. The formula of AGR is as follows:

1 $$ \mathrm{AGR}=\sqrt[n]{\frac{B}{A}}-1 $$

where B is the quantity of the five types of health-care resource in 2016, A is the quantity of the five types of health-care resource in 2010, and n represents the number of years. We used AGR (Average Growth Rate) instead of GR (Growth Rate) to measure the time trends of health-care resources for the advantages of its accuracy to calculate historical tracks and comparability of the relative performance of the health-care resource allocation.

There exist many measures for assessing the equity of health-resource allocation, such as the Gini coefficient, Lorenz curve and Theil index. Among them the Theil index is primarily used statistically to measure income inequality or other economic phenomena between different individuals or within varied groups. It is a special case of the generalized entropy index and one of the most widely used measures of inequality in regional economic development. The Theil index was initially proposed by an econometrician named Henri Theil at Erasmus University Rotterdam [16], and it is often formulated as follows:

2 $$ T=\frac{1}{n}\;\sum \limits_{i=1}^n\frac{y_i}{\overline{y}}\;\log\;\left(\frac{y_i}{\overline{y}}\right), $$

where *T* is the Theil index, which means income allocation inequality, and *y*_*i*_ and $\overline{y}$ represents the income of individual *i* and the average income of the population, respectively.

The Theil index can be calculated with another form to measure the inequality between different groups, which is known as the between-region difference. This formula can be written as follows:

3 $$ T=\sum \limits_{i=1}^k{w}_i\ln \left(\frac{w_i}{e_i}\right), $$

where *w*_*i*_ represents the proportion of the income of group *i* accounting for the total income of all groups and *e*_*i*_ represents the proportion of the people in group *i* accounting for the overall population of all groups. Here in our study we defined *w*_*i*_ as the proportion of health-care resources in district *i* accounting for the resources of the Shanghai city, and defined *e*_*i*_ as the proportion of the people in district *i* accounting for the overall population of the city. The value of the Theil index ranges from 0 to 1, with zero representing perfect equality and one meaning completely unequal.

Statistical analyses were performed using Stata statistical software version 15.0 (StataCorp LP, College Station, TX, U.S. [17], and maps were generated using ArcGis 10.6 (Environmental Systems Research Institute, Redlands, CA, USA) [18].

## Results

### Differences in geographic distribution of health-care resource allocation at the city and its division level in Shanghai from 2010 to 2016

The descriptive statistics of indicators of health-resource allocation in Shanghai can be seen from Table 2, and changes in numbers and AGRs of health-resource allocation in Shanghai are presented in Table 3. To visually display the data distribution through their quartiles, we use whiskers box plot to interpret the changes of each indicator of health-care resource allocation per every year from 2010 to 2016 (see Fig. S1 in the additional file). Seen from the tables, the health-care resources increased gradually from 2010 to 2016, the quantities of health-care resources per 1000 population all increased, the ratios of health institutions, beds, technicians, doctors and nurses per 1000 population rose by 6.99, 2.67, 3.81, 3.30 and 5.13% annually from 2010 to 2016, respectively. The number of institutions grew faster than the health workforce did overall, and the number of beds per 1000 people grew the slowest among the five types of indicators. For example, from 2010 to 2016, the number of institutions per 1000 people increased by 6.99% annually, the numbers of technicians, doctors and nurses per 1000 people annually increased by 3.81, 3.30 and 5.13%, respectively, while the number of beds rose by 2.67%, only accounting for 3/8 of AGR of the institution indicators during the same period (Table 3).

**Table 2:** Descriptive Statistics of indicators of health-resource allocation in Shanghai (2010–2016; per 1000)

Indicators	Obs.	Min.	Max.	Mean.	Median
Number of institutions	112	0.07	0.51	0.21	0.17
Number of beds	112	2.05	17.52	5.98	4.59
Number of technicians	112	3.08	28.08	8.20	5.63
Number of doctors	112	1.23	10.03	3.02	2.08
Number of nurses	112	1.16	12.92	3.58	2.26

**Table 3:** Changes in the numbers and AGRs related to health-resource allocation in Shanghai (2010–2016; per 1000)

Indicator	2010	2011	2012	2013	2014	2015	2016	AGR
Number of institutions	0.14	0.14	0.15	0.20	0.21	0.21	0.21	6.99%
Number of beds	4.56	4.56	4.60	4.73	4.84	5.08	5.34	2.67%
Number of technicians	5.88	5.92	6.14	6.47	6.76	7.05	7.36	3.81%
Number of doctors	2.23	2.22	2.28	2.40	2.53	2.61	2.71	3.30%
Number of nurses	2.43	2.51	2.66	2.81	2.97	3.12	3.28	5.13%

*AGR* Average Growth Rate

**Fig. 1:**
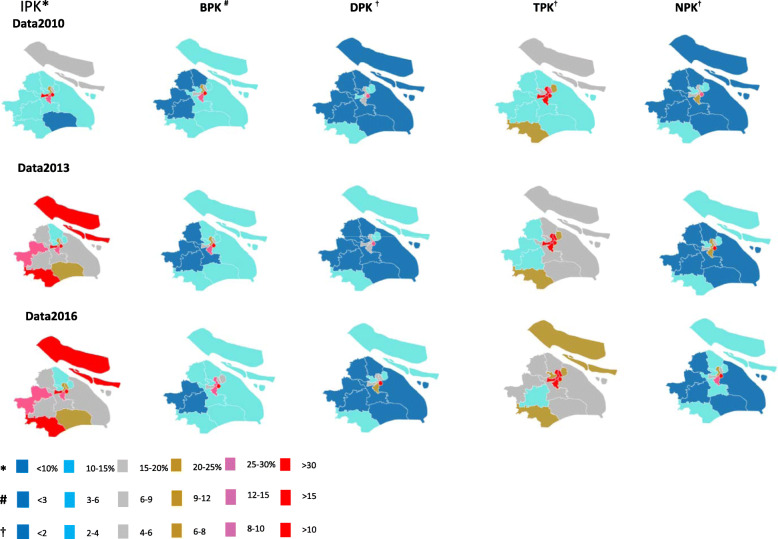
Time Trends and Geographic Distribution in the Health-care Resource Allocation Across all Shanghai’s Districts From 2010 to 2016.IPK: Number of institutions per 1000 people; BPK: Number of beds per 1000 people; DPK: Number of doctors per 1000 people; TPK: Number of technicians per 1000 people; NPK: Number of nurses per 1000 people

Figure 1 showed the time trends and geographic distribution of the health-care resource allocation across all Shanghai’s districts from 2010 to 2016 (for space constraints, only data from 2010, 2013 and 2016 were presented in Fig. 1). As for administrative divisions, from 2010 to 2016, an overall increasing trend was observed in the numbers of institutions, beds and workforce per 1000 people across all districts, meanwhile with central districts with darker red indicating having the highest health-care resource densities than suburban districts with darker blue indicating the lowest density. This indicated an unchanged distribution concentration both in health-care facility and workforce resource allocation among urban areas other than rural ones in Shanghai. However, among the different types of health-care resource, central and suburban districts grew differently during the same period. Compared with suburban districts, central districts grew faster in the AGRs of the beds and nurses per 1000 population and increased slower in the AGRs of the institutions, technicians and doctors per 1000 population. Notedly, some central and suburban districts even reversely experienced a negative average growth during this period.

**Fig. 2:**
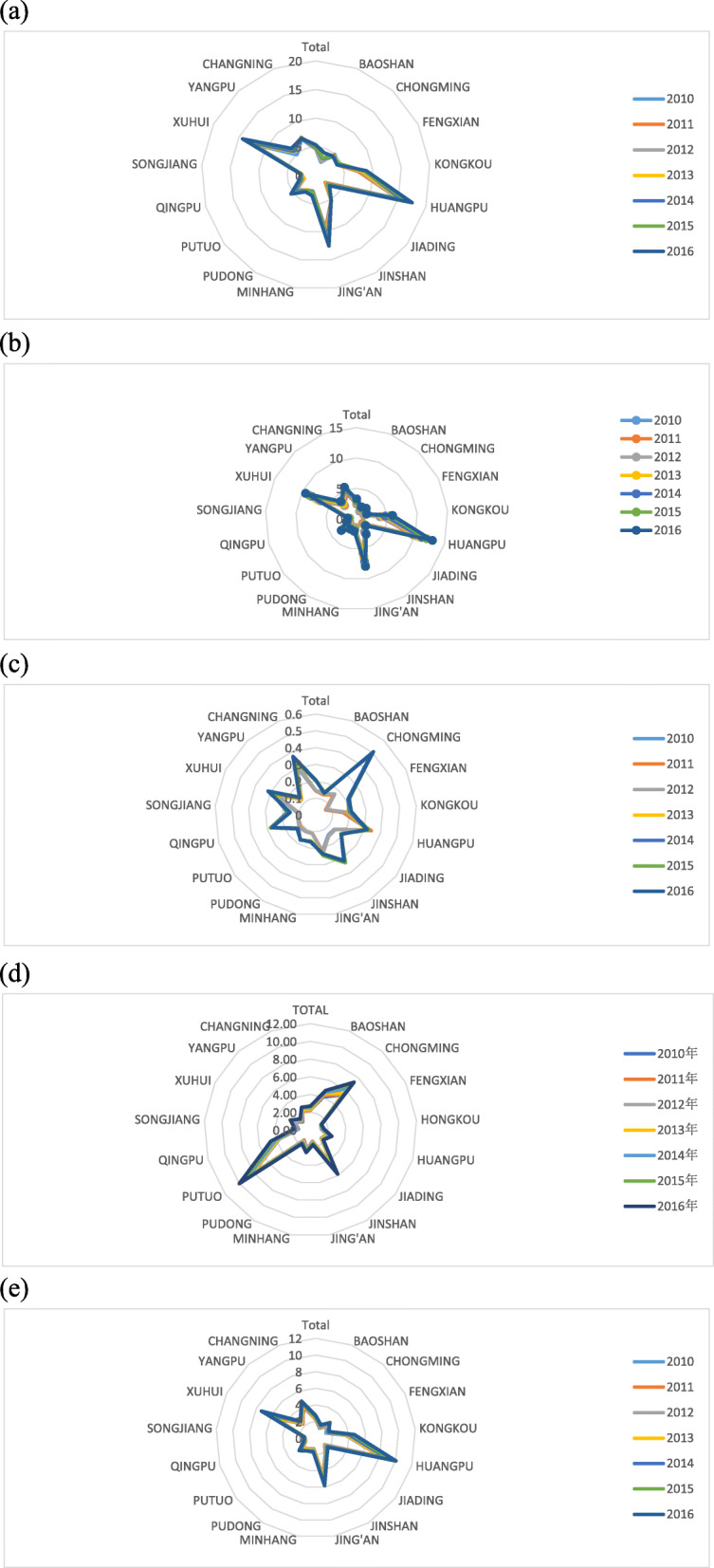
Per 1000 Health Resources Across the Districts in Shanghai From 2010 to 2016 **a** presents per 1000 beds across the districts in Shanghai from 2010 to 2016; **b** presents per 1000 nurses across the districts in Shanghai from 2010 to 2016; **c** presents per 1000 health institutions across the districts in Shanghai from 2010 to 2016; **d** presents per 1000 technicians across the districts in Shanghai from 2010 to 2016; **e** presents per 1000 doctors across the districts in Shanghai from 2010 to 2016

Figure 2a and b present the quantities of beds and nurses per 1000 population, respectively, across all the districts from 2010 to 2016. While the total amount of health resources was observed increasing across all the districts, there was a wide gap between central and suburban districts in the ratios and average growth rates of these two indicators. Compared with suburban areas, central ones had higher ratios of beds and nurses per 1000 population. From 2010 to 2016, we observed an increasing trend in the number of beds per 1000 population across mostly of districts, especially for main central district Huangpu, Jing’an, and Xuhui, rising from 15.10 to 17.52, 9.38 to 12.57 and 12.23 to 14.29, respectively. Among these districts, Huangpu had the biggest average ratio in 2016, with above 17 beds per 1000 people. Jing’an and Xuhui were also above 10 in this ratio that year, higher than other districts, demonstrating higher bed density in the central areas of Shanghai. Reversely, that indicator of Chongming and Fengxian, which were located far from central areas of Shanghai, decreased noticeably from 4.94 to 4.52,4.20 to 4.13 in the same year, respectively. A similar trend was also observed in the number of nurses per 1000 population during the period.

Figure 2c, d and e illustrate the numbers of institutions, technicians and doctors per 1000 population, respectively, across Shanghai’s districts from 2010 to 2016. Though suburban districts occupied fewer average ratios of the institutions, technicians and doctors than urban ones, they were observed growing faster annually in these indicators during the period. For example, from 2010 to 2016, Fengxian, Chongming, Qingpu, and Jinshan were the fastest fourth of annually growth among other administrative divisions, with the AGRs of 22.39, 21.15, 16.14 and 14.79%, respectively. Aside from AGRs, each of these four suburban districts also had higher ratios of the institutions, technicians and doctors than other suburban districts, an even some central districts. For example, in 2016, Chongming district had 0.51 institutions per 1000 people, more than twice of the average number of institutions for central districts in the same year. It was also noticed that Huangpu reversely experienced a negative average growth rate of − 0.68%, with decreasing from 0.33 in 2010 to 0.32 in 2016. It was possibly due to this new district’s administrative division merging in 2011, resulting in the decease of total number of health institutions. Similarly, both the number of technicians and doctors per 1000 people showed the same trend as that observed for the number of institutions per 1000 people.

### Inequality in health-resource allocation at the whole city level in Shanghai from 2010 to 2016

**Fig. 3:**
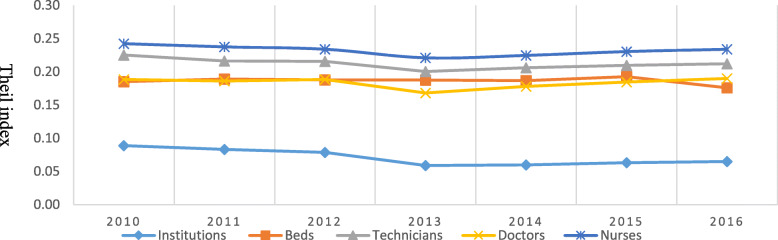
Trends of The Theil Indexes For the Health Resources in Shanghai From 2010 to 2016

Table 4 and Fig. 3 present the Theil indices of health-resource allocation in Shanghai from 2010 to 2016. When vertically comparing data from the same year, the Theil indices of the technicians, doctors, nurses and beds were all greater than that for institutions. This indicated the existence of unbalance of workforce and bed allocation in Shanghai during this period. For example, in 2016, the Theil indices of the numbers of technicians, doctors, nurses and beds were 0.21, 0.23, 0.20 and 0.18, respectively, whereas the index of institutions was 0.06. Similar unbalance also existed in other 6 years from 2010 to 2015.

**Table 4:** Theil indices related to health-resource allocation in Shanghai (2010–2016)

Indicators	2010	2011	2012	2013	2014	2015	2016
Number of institutions	0.09	0.08	0.08	0.06	0.06	0.06	0.06
Number of beds	0.19	0.19	0.19	0.19	0.19	0.19	0.18
Number of technicians	0.23	0.22	0.22	0.20	0.21	0.21	0.21
Number of doctors	0.19	0.19	0.19	0.17	0.18	0.18	0.20
Number of nurses	0.24	0.24	0.23	0.22	0.22	0.23	0.23

As for Theil index trends for health-care resources in Shanghai from 2010 to 2016, the indices of all indicators exhibited a decline trend, except for the numbers of doctors. This indicated a reduction in the inequality with respect to most health-care resource indicators in Shanghai over the 7 years. For example, the Theil index for institutions decreased from 0.09 in 2010 to 0.06 in 2013, followed by a little increase to 0.06 in 2016. The Theil index of beds also declined from 0.19 in 2010 to 0.18 in 2016. The trend in Theil index for technicians and nurses were the same as that of institutions, with a decrease from 2010 to 2013 and then an increase until the period end. This range was 0.23–0.21 for the technicians, and 0.24–0.23 for the nurses, respectively. This indicated a reduction in the inequality of these two types of health-care workforce in that period.

However, for the Theil indices of the doctor, reverse trends were observed in the numbers of doctors during this period. For example, there was an overall increase trend over 7 years, from 0.19 in 2010 to 0.20 in 2016, which demonstrated that the problem of inequality of doctor allocation in Shanghai had not been solved.

## Discussion

This study analyzed the temporal trends and inequality of health-resource allocation at district level in Shanghai, noting trends of improvements in the quantity and inequality in health-resource allocation from 2010 to 2016. However, various regions were noticed to have unbalanced distributions of health workforce, especially for doctors, which exhibited serious inequalities in either number or temporal trend.

Firstly, this study observed that the number of institutions, the number of beds, the number of technicians, number of doctors, the number of nurses all increased over the 7 years. These results made obvious that the China’s government’s targets of reforming the health-care system to operate smoothly, and providing a safe, efficient, and convenient health service over past 7 years have been achieved. To expand, utilize and optimize health-care resources on the supply-side, according to the “Healthy China 2030” plan, China has strengthened health subsystems by financially investing in health institutions to purchase and supplement beds, equipment and other facilities; recruit and educate technicians and doctors; make health institutions function reoriented; update the health-care service model based on the status of public health; and provide a collaborative hierarchical health-care system that meets people’s medical demands [19–22]. This included not only perfecting plans for the geographical distribution of health-care resources across different regions and districts [23], but also making a dynamic balance in allocation between facility and workforce. On the demand-side, the government has educated Chinese people about the “big health” concept to promote healthy lifestyles, as well as re-designed medical insurance to widen coverage among poorer people [24], under which more and more patients were given reasonable access to health-care resources. Thereby, the aforementioned efforts that the Chinese and Shanghai governments made have caused increased numbers of institutions, beds, technicians, doctors, and nurses across different districts, and also reduced the inequality in health-care resource allocation from 2010 to 2016. Numerous studies have reached similar results [25–28].

Secondly, this study observed regional differences in health-resource distribution at district level from 2010 to 2016. Health programs were unbalanced in their development when central and suburban districts were compared, which resulted in urban-rural regional disparity in health-care resources. This was due to some historical reasons as followed. Most of public hospitals, especially tertiary hospitals were originally distributed in urban or municipal districts. They received more supports including fiscal reimbursement and policy inclination from Shanghai government than those located in rural districts. Furthermore, due to the new 2009 reform initiatives to strengthen the status of public hospital, plus the improved transportation convenience and accessibility to health care, the institutions located in urban districts were overwhelmed in term of beds, equipment and other facilities, forming a Matthew Effect that, the institutions in urban districts were visited by more and more people, while they were more financially invested by the government. Contrastedly, the institutions in rural areas were under-developed to form the socio-economic and health-care accessibility gaps between urban and suburban districts. Some relevant studies have also noted the distribution imbalance of institutions [27, 29, 30]. Furthermore, this effect also led to increasing numbers of the health workforce being attracted from suburban to central districts for pursuing higher salary, prospective career development in urban hospitals. We also found that some suburban districts grew faster than urban ones did in the numbers of institution, technician and doctor in light of AGRs. This was possibly attributed to some measures taken by Shanghai government to implement the strategy of “5 + 3 + 1” since 2009, which aimed to multiple the numbers of the tertiary hospitals and their workforce, equipment and other facilities in rural districts, in order to narrow the public hospital distribution gap between the urban and suburban districts and increase health care accessibility for residents of rural districts. For example, Chongming, Fengxian, and QingPu district were referred to the “3” sub-strategy targets of “5 + 3 + 1” public hospital reform to update their public hospitals into tertiary hospitals to meet the demands of health-care in these three administrative divisions since 2009. This result on the regional difference is similar to those of studies that discovered an overcentralized health-care resource in urban areas and rapidly growing numbers of institution and workforce items in suburban areas in China [31–34].

Third, this study used the Theil index to examine inequality in health-care resources allocation. Though the index has some disadvantages, such as being complicated to calculate and interpret; a wide variety when distribution varies regardless of the change that occurs in the top, middle, or bottom tier of resources; and the fact that when contrasting populations with different sizes, the calculation is dependent on the number of individuals in the population or group, this measurement method can bring about robustness when determining inequality within and between separate groups, with high sensitivity to the efficiency of health-care resource allocation. This is because the index is decomposable by group components, can incorporate group-level data, and is particularly effective at paring effects in hierarchical data sets [35]. This study demonstrated the inequality among technicians, doctors, nurses, beds and institutions from 2010 to 2016. On one hand, the Theil indices of workforce and beds were higher than that of institutions in Shanghai when comparing same year’s data, demonstrating unbalanced distribution of health-care resources between institutions and workforce. This is attributable to the fact that, compared with expanding health institutions, recruiting and educating health workforce is a longer-term work to narrow the health-care resource distribution gap between urban and suburban districts. As mentioned above, technicians, doctors and nurses were more likely to work in bigger institutions in urban districts, given the relative advantages of salary and career development. Accordingly, for rural districts, it was harder to quickly attract or educate health workforce in a short time period based on condition of lower salary and socio-economic constrains, whereas expanding and opening branch institutions and facilities were easier to achieve. This result is consistent with the findings reported by Chen R. et al. [12], Liang D. et al. [36], and Li D. et al. [37]. On the other hand, the Theil index of doctors, increased during this period, indicating worsening inequality in physician allocation. The possible reason could be that the elevated provision of human resources does not necessarily indicate a decline in inequity, as has been noted in other countries [38–41], or increasing numbers of rural doctors flow into larger urban tertiary institutions. Another reason is distribution disparity among different institutions. Perhaps Shanghai has developed a certain cultural tradition that people are more likely to choose to work in public sectors, such as government-affiliated sectors or public hospitals, resulting in the underdevelopment of private hospital sectors. Therefore, doctors with good quality had more probability of being employed in famous or public health institutions, while ones with not-good quality had to be in nonfamous or private health institutions, further exacerbating the disparities between doctors at different levels. This finding was similar to those of some relevant studies, which have confirmed the physician distribution gap among different regions or hospitals of various sizes [42–45].

Some caveats of the study should be noted. Firstly, the data used could possibly only reflect the status of the health-resource allocation in Shanghai at the cut-off point, as we could only obtain them from the Chinese Yearbooks by Chinese authorized publisher at least 2 years after the year the data were collected. Therefore, critical information may have been omitted from our data. A future study on changes in health-care resource allocation from 2017 to the present, along with comparisons with the present study, can be carried out when the data are available. Secondly, this study did not take consideration of the impact of the health outcomes in the population on health-care resource allocation. According to the health capacity paradigm theory (HCP) proposed by Chakraborty and Chakraborti [46], the population’s health status in one region will have mutual effects on health-care resource allocation in that area. Due to time constraints, we investigated the one-way impacts without consideration of these factors, which may have somehow affected the results. Thirdly, we selected indicators for health-resource allocation instead of indicators of the quality of health services. Factors represented by other unmeasured indicators may have influenced the current results. Thus, integrating the indicators used for health-resource allocation in this study with those for health service quality may yield more robust results in a future study.

## Conclusion

Health-care resources increased and inequality in health resource allocation decreased in Shanghai from 2010 to 2016. This confirms the measures taken by the Chinese government since 2009 when starting health care reform, specifically in regard to institutions, technicians, doctors in Shanghai are successful. However, the distribution of health-care resources made a difference between urban and rural areas, and inequality of workforce, especially doctor, had not been mitigated until 2016 since 2009’s health care reform, demonstrating that the measures taken by the government to improve the attractivity of health institutions in rural areas and increase health care accessibility for residents there were needed to be strengthened further. Therefore, to achieve a regional balance in health-care resource distribution between central and rural areas and improve the equality of workforce allocation in Shanghai, policies should not pay attention to improve the socioeconomic levels in rural districts by raising income, make transportation conditions better, invest more fiscal funds and providing more access to health-care service for residents of rural districts. They should also focus on the balance of physician distribution between institutions at various levels, such as remobilization, job performance evaluation. To further investigate health-resource allocation, a prospective study will be carried out to combine the indicators used in this study with ones of health service quality.

## Supplementary Information

**Additional file 1: Fig. S1.** Changes for each indicator with whiskers box plot per every year from 2010 to 2016(per 1000). (a) presents the number of institutions per 1000 people in Shanghai; (b) presents the number of beds, technicians, doctors and nurses per 1000 people in Shanghai.

## Data Availability

The data analyzed during the current study are available from the corresponding author on reasonable request.

## Abbreviation

AGR: Annual growth rate

## References

[1] i Casasnovas GL, Rivera B, Currais L, editors. Health and economic growth: findings and policy implications. US: Mit Press; 2005.

[2] Cutler DM, Lleras-Muney A, Vogl T. Socioeconomic status and health: dimensions and mechanisms. US: National Bureau of Economic Research; 2008.

[3] Culyer AJ, Wagstaff A (1993). Equity and equality in health and health care. J Health Econ.

[4] Tao Y, Henry K, Zou Q (2014). Methods for measuring horizontal equity in health resource allocation: a comparative study. Health Econ Rev.

[5] Szalai J (1986). Inequalities in access to health care in Hungary. Soc Sci Med.

[6] Chen LY, Chen SY. “1560” medical circle has basically built in Shanghai, and at least one tertiary hospital is available in each district. http://district.ce.cn/newarea/roll/201212/28/t20121228_23983925.shtml. Accessed 28 Dec 2012.

[7] Cheng TM (2013). Explaining Shanghai’s health care reforms, successes, and challenges. Health Aff.

[8] Bao Y, Du XL, Zou LM (2010). Analysis and policy suggestion of hospitalizing intension of residents in Shanghai. J Shanghai Jiaotong Univ Med Sci.

[9] Wang S, Xu J, Jiang X (2018). Trends in health resource disparities in primary health care institutions in Liaoning Province in Northeast China. Int J Equity Health.

[10] Sun J (2017). Equality in the distribution of health material and human resources in Guangxi: evidence from southern China. BMC Res Notes.

[11] Sun J, Luo H (2017). Evaluation on equality and efficiency of health resources allocation and health services utilization in China. Int J Equity Health.

[12] Chen R, Zhao Y, Du J, Wu T, Huang Y, Guo A (2014). Health workforce equity in urban community health service of China. PLoS One.

[13] Pan J, Shallcross D (2016). Geographic distribution of hospital beds throughout China: a county-level econometric analysis. Int J Equity Health.

[14] Zhang Y, Wang Q, Jiang T, Wang J (2018). Equity and efficiency of primary health care resource allocation in mainland China. Int J Equity Health.

[15] Fan X, Zhang D, Li X (2018). Distribution equality of mental health facilities and psychiatric beds in Shanghai based on Theil index. Chin Ment Health J.

[16] Theil H (1967). Economic and information theory.

[17] StataCorp 15 (2014). Stata survey data reference manual, release 12.

[18] ESRI (2011). ArcGIS desktop: release 10.

[19] Kuang L (2012). Strategy for optimizing the mechanism of healthcare competition: establishing the vertical integrated healthcare delivery systems. Chin J Health Policy.

[20] Dan-dan Z (2008). The reality and preliminary analysis on medical resource and their vertical integration in Shanghai. Chin Health Resour.

[21] Iriart C, Merhy EE, Waitzkin H (2001). Managed care in Latin America: the new common sense in health policy reform. Soc Sci Med.

[22] Barr DA. Introduction to US health policy: the organization, financing, and delivery of health care in America. US: JHU Press; 2016.

[23] Wenjie R (2014). The path selection of medical resource optimizing allocation. Chin Health Resour.

[24] Hai QS, Jin YJ (2017). Connotation and basic characteristics of great health concept. J Tradit Chin Med.

[25] Dan-dan Z (2008). The realty and preliminary analysis on medical resource and their vertical integration in Shanghai. Chin Health Resour.

[26] Zhang LF, Li LQ (2019). Study on the equilibrium of spatial allocation of medical resources at different levels in Shanghai. Urban Dev Stud.

[27] Zhang LF. Study on the equilibrium and optimization of medical resource allocation in metropolitan areas: a case study of Shanghai. Nanjing J Soc Sci. 2019;(02):65–72 (in Chinese).

[28] Wang D, Ma L, Gong EJ (2016). Analysis of the dominant diseases of tertiary public hospitals newly built in suburbs in Shanghai. Chin Hosp Manag.

[29] Shen WW, Bao Y (2015). Analysis of nursing service needs of old people in Shanghai nursing institutions for the aged and policy recommendations. J Shanghai Jiaotong Univ Med Sci.

[30] Shi J, Tang L, Jing L, Geng J, Liu R, Luo L, Chen N, Liu Q, Gong X, Bo X, Yang Y, Wang Z (2019). Disparities in mental health care utilization among inpatients in various types of health institutions: a cross-sectional study based on EHR data in Shanghai, China. BMC Public Health.

[31] Dong EH, Li GH, Cai YY (2016). A review on regional difference in healthcare resource allocation. Chin Health Resour.

[32] Fan XA. The lag of medical reform affects the effectiveness of medical insurance, and it is the key for doctors to go to the grassroots level. China Health Insur. 2014;(12):23–4 (in Chinese).

[33] Gong SW, Li ZG, Xu Y, et al. Research on regional distribution differences of healthcare resources in China from a perspective of accessibility. Chin J Hosp Adm. 2011;(05):325–30 (in Chinese).

[34] Xu Z, Geng H, Zeng Z. The analysis of medical resources allocation between Nanjing third-level hospitals and community health center. Chin Health Serv Manag. 2012;(05):335–8 (in Chinese).

[35] Tao Y, Henry K, Zou Q, Zhong X (2014). Methods for measuring horizontal equity in health resource allocation: a comparative study. Health Econ Rev.

[36] Liang D, et al. Does rapid and sustained economic growth lead to convergence in health resources: the case of China from 1980 to 2010. Inquiry (United States). 2016;53:1-6. 10.1177/0046958016631699 PMC5798700 26895881

[37] Li D, et al. Unequal distribution of health human resource in mainland China: what are the determinants from a comprehensive perspective? Int J Equity Health. 2018;17(1):1-12. 10.1186/s12939-018-0742-z PMC5830142 29486791

[38] Paraje G, Vásquez F (2012). Health equity in an unequal country: the use of medical services in Chile. Int J Equity Health.

[39] Glorioso V, Subramanian SV (2014). Equity in access to health care services in Italy. Health Serv Res.

[40] Smith S, Normand C (2011). Equity in health care: the Irish perspective. Health Econ Policy Law.

[41] Ghosh S (2014). Equity in the utilization of healthcare services in India: evidence from national sample survey. Int J Health Policy Manag.

[42] Zou YJ (2010). Comments on brain drain in private hospitals of China. Jiangsu Healthc Adm.

[43] Zhang XJ, Zhu K (2018). Equity in the distribution of human resources for health in China during 2004-2015. Chin Gen Pract.

[44] Anand S, Fan VY, Zhang J, Zhang L, Ke Y, Dong Z, Chen LC (2008). China’s human resources for health: quantity, quality, and distribution. Lancet.

[45] Zhu B, et al. Detecting the priority areas for health workforce allocation with LISA functions: an empirical analysis for China. BMC Health Serv Res. 2018;18(1):1-14. 10.1186/s12913-018-3737-y PMC6292090 30541543

[46] Chakraborty R, Chakraborti C (2015). India, health inequities, and a fair healthcare provision: a perspective from health capability. J Hum Dev Capab.

