# Supplementary material for: Differences in regional distribution and inequality in health-resource allocation on institutions, beds, and workforce: a longitudinal study in China
Source: Arch Public Health. 2021 May 17;79:78. doi: 10.1186/s13690-021-00597-1 (PMC8130126; doi:10.1186/s13690-021-00597-1)
Supplement: Supplementary file 1 — Additional file 1: Fig. S1. Changes for each indicator with whiskers box plot per every year from 2010 to 2016(per 1000). (a) presents the number of institutions per 1000 people in Shanghai; (b) presents the number of beds, technicians, doctors and nurses per 1000 people in Shanghai. [file 13690_2021_597_MOESM1_ESM.docx]

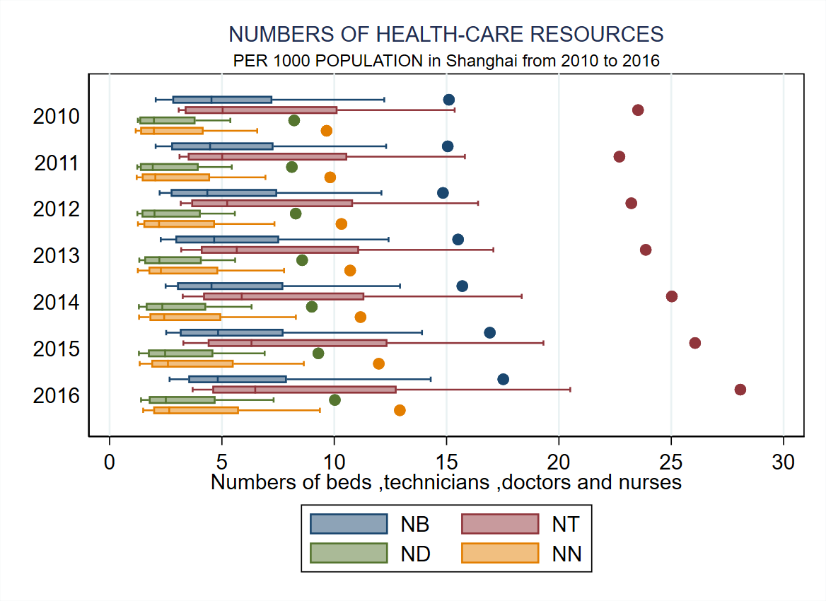


YEAR

YEAR

(a) (b)

Fig.S1 Changes for each indicator with whiskers box plot per every year from 2010 to 2016(per 1000).

(a) presents the number of institutions per 1000 people in Shanghai; (b) presents the number of beds, technicians, doctors and nurses per 1000 people in Shanghai.
